# Gut Microbiota and Autism Spectrum Disorder: A Neuroinflammatory Mediated Mechanism of Pathogenesis?

**DOI:** 10.1007/s10753-024-02061-y

**Published:** 2024-08-02

**Authors:** Fatemeh Zarimeidani, Rahem Rahmati, Mehrnaz Mostafavi, Mohammad Darvishi, Sanaz Khodadadi, Mahya Mohammadi, Farid Shamlou, Salar Bakhtiyari, Iraj Alipourfard

**Affiliations:** 1 Students Research Committee, Shahrekord University of Medical Sciences, Shahrekord, Iran; 2 Faculty of Allied Medicine, Shahid Beheshti University of Medical Sciences, Tehran, Iran; 3 School of Aerospace and Subaquatic Medicine, Infectious Diseases & Tropical Medicine Research Center (IDTMC), AJA University of Medical Sciences, Tehran, Iran; 4 Student Research Committee, Tehran Medical Sciences Branch, Islamic Azad University, Tehran, Iran; 5 Student Research Committee, School of Medicine, Shahid Beheshti University of Medical Sciences, Tehran, Iran; 6 School of Medicine, Isfahan University of Medical Sciences, Isfahan, Iran; 7 Feinberg Cardiovascular and Renal Research Institute, North Western University, Chicago. Illinois, USA; 8 Institute of Physical Chemistry, Polish Academy of Sciences, Marcin Kasprzaka 44/52, 01-224, Warsaw, Poland

**Keywords:** autism spectrum disorder, biomarker, gut microbiota, inflammation, neuroinflammation

## Abstract

Autism spectrum disorder (ASD) is a neurodevelopmental disorder characterized by impairments in social communication and behavior, frequently accompanied by restricted and repetitive patterns of interests or activities. The gut microbiota has been implicated in the etiology of ASD due to its impact on the bidirectional communication pathway known as the gut-brain axis. However, the precise involvement of the gut microbiota in the causation of ASD is unclear. This study critically examines recent evidence to rationalize a probable mechanism in which gut microbiota symbiosis can induce neuroinflammation through intermediator cytokines and metabolites. To develop ASD, loss of the integrity of the intestinal barrier, activation of microglia, and dysregulation of neurotransmitters are caused by neural inflammatory factors. It has emphasized the potential role of neuroinflammatory intermediates linked to gut microbiota alterations in individuals with ASD. Specifically, cytokines like brain-derived neurotrophic factor, calprotectin, eotaxin, and some metabolites and microRNAs have been considered etiological biomarkers. We have also overviewed how probiotic trials may be used as a therapeutic strategy in ASD to reestablish a healthy balance in the gut microbiota. Evidence indicates neuroinflammation induced by dysregulated gut microbiota in ASD, yet there is little clarity based on analysis of the circulating immune profile. It deems the repair of microbiota load would lower inflammatory chaos in the GI tract, correct neuroinflammatory mediators, and modulate the neurotransmitters to attenuate autism. The interaction between the gut and the brain, along with alterations in microbiota and neuroinflammatory biomarkers, serves as a foundational background for understanding the etiology, diagnosis, prognosis, and treatment of autism spectrum disorder.

**Graphical Abstract:**

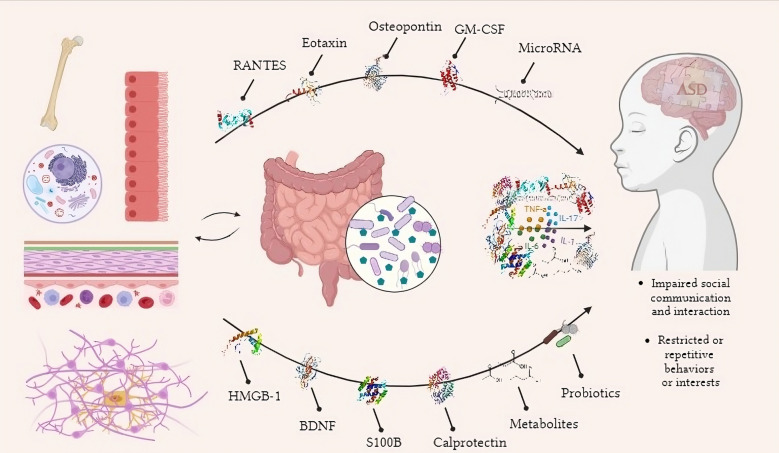

## Introduction

The human gut microbiota is a complex biome and variable collection of microorganisms interacting with one another and the human host, including bacteria, fungi, archaea, and viruses. The gut microbiome influences many aspects of host health, including immune system control, gut hormone regulation, and neuronal transmission. It modifies the ingested medications and their metabolism, toxin clearance, and the generation of numerous host-affecting agents [1]. The gut microbial load can directly or indirectly influence the brain via a mutual relationship known as the "gut-brain axis." The gut microbiota can directly influence the brain by producing neuroactive substances such as neurotransmitters, amino acids, and microbial metabolites. These substances can potentially interfere with the host immune system and metabolism, affecting the gastrointestinal (GI), nervous system and vagus nerve. The gut microbiota can also influence the integrity of the gut barrier, which limits the passage of luminal substances into the bloodstream. Accessibility of such bacterial structural components like lipopolysaccharides or by-products of metabolic activities like short-chain fatty acids (SCFAs) may result in an inflammatory cascade that affects the CNS [2].

Autism spectrum disorder (ASD) is a persistent psychological abnormality characterized by impaired social communication and limiting and repetitive behavior patterns, hobbies, or activities [3]. Both genetic and environmental variables have been involved in ASD. Recent studies have shown that inflammation and inflammatory mediators have a role in disease genesis. Inflammatory elements that contribute to ASD include unusual microglia activation and polarization phenotypes, higher systemic levels of pro-inflammatory mediators, and altered patterns of immune cell responsiveness to activation triggers [4].

Numerous types of research in recent years have implicated gut bacteria in the etiology of ASD. However, studies have found that the structure of the gut microbiota is meaningfully changed in ASD; the significance of the gut microbiota as an etiology of ASD is yet unclear. It has been accepted that the microbiome of autistic children differs from healthy individuals [4]. Inflammatory deviations are potential etiology candidates in how gut microbiota can influence the gut-brain axis of ASD patients. Neuroinflammatory factors in ASD result from changes in the regulation of intestinal barriers, activation and function of microglia, and levels of neurotransmitters [5, 6].

ASD is currently diagnosed based on clinical symptoms, which can lead to delays and misinterpretation. Biomarkers based on neuroinflammatory processes associated with gut microbiota may provide a more objective and precise way of detecting ASD. Several examples of these markers include microRNAs that modulate immune signaling; brain-derived neurotrophic factor (BDNF), which promotes brain growth; S100B, which reflects neural immunity; and chemokines that facilitate immunological activation, such as RANTES and eotaxin [7]. This review aims to evaluate and discuss neuroinflammatory biomarkers in the pathogenesis and potential diagnostic trials of ASD in more detail.

## Gut Microbiota, Inflammation, and ASD

Nearly two decades earlier, a potential association between gut microbiota and ASD was proposed [8]. While the exact cause of ASD is still unknown, existing literature has shown that gut dysbiosis, along with a neuroinflammatory condition, is found in individuals with ASD [9]. Studies found inconsistent differences in the gut microbiota composition of ASD cases. Overall, the ASD population shows signs of dysbiosis, with a different abundance of *Bacteroidetes/Firmicutes, Prevotella, Clostridium*, *Lactobacillus, Bifidobacterium, Faecalibacterium, Streptococcus, Enterobacteriaceae, Verrucomicrobia, Fusobacteria, Escherichia coli, Enterococcus, Akkermansia, Phascolarctobacterium*, and lots of other microbes compared to healthy controls [10–12]. Nonetheless, specific microbial patterns associated with ASD remain unidentified.

Moreover, Cuomo et al. [9] recently indicated that gut dysbiosis and inflammation were identified by host fecal DNA-specific methylation in autistic children. They revealed that autistic patients with dysbiosis significantly enriched inflammatory and immune pathways, including the production of interleukin (IL)-2, 6, and 12 and the activation of the toll-like receptor (TLR) 3 signaling pathway. Consistently, several studies corroborated earlier findings of the neuroinflammation caused by dysbiosis in various neurodegenerative and neuropsychiatric conditions [12, 13]. Disruptions in immune signaling pathways like the NLRP3 inflammasome, type 1 interferon, and nuclear factor kappa-light-chain-enhancer of activated B cells (NF-κB) signaling pathways are among the possible dysbiotic consequences. Alterations to the T-helper 17 cell/T-reg proportion and imbalances in macrophage polarization, tumor necrosis factor (TNF)-α, IL-1β, 18, and 6 are also possible [14, 15].

On the other hand, the involvement of inflammation and immunological dysregulation has been indicated in the development and/or severity of ASD [16, 17]. Prior investigations on autistic cases have shown elevated levels of inflammatory markers such as TNF-α, interferon-γ, IL-2,4,5,6,8,17, and 10 [12, 18, 19]. A study also reported that autistic children with an innate proinflammatory response or impaired T cell activation indicate more severe behavioral issues compared to those with noninflamed or non-T cell-activated immunological profiles [20]. Given the lack of research on gut microbial composition and metabolites in autistic patients concerning inflammatory conditions (Table 1), the precise relationship between these three factors remains unclear and requires further investigation. Overall, gut microbiota seems to play a crucial role in ASD through inflammation.

**Table 1:** Studies on the association of neuroinflammatory biomarkers with microbial alternation and autistic behaviors

Author (year)	Population	Biomarker	Method	Microbial alteration	Association with behavioral symptoms
Abuaish (2021) [21]	28 Sprague Dawley male rats	BDNF	qRT-PCR	Fecal transmission of *Bifidobacterium* balanced the fecal *Clostridium* spp. and normalized the level of BDNF expression.	Association between the impairment in social behavior and augmented BDNF transcript levels in the hippocampus
Chen (2020) [22]	C57BL/6N mice	RANTES and Eotaxin	Th1/Th2 and Chemokine 20- Plex Mouse Procarta Plex™ Panel 1 and the Bioplex200 system	Negative correlation between *Clostridiaceae, Erysipelotrichaceae, Prevotella_other*, *Candidatus Arthromitus* and *Proteus* with serum levels of RANTES and Eotaxin.	Improved anxiety-like and repetitive behaviors in mice with gut microbiota transplantation
Carissimi [2019] [23]	30 ASD children and 14 controls	HMGB-1	Western blot stool	• ↓ Gut microbiota biodiversity • Under-representation in the gut microbiota of ASD subjects of several functions, such as catabolism of 3,3 phenylpropionate • Loss of *E. coli* strains known to regulate the propionate catabolism	Relationship between fecal HMGB1 levels and severity of GI symptoms
Iovene (2017) [24]	47 ASD and 33 healthy children	Calprotectin	ELISA	↑ Candida spp. and ↓ Lactobacillus spp.in ASD patients	• Correlation between disease severity (CARS score) and calprotectin and Clostridium spp. presence • Correlation of GI symptoms, such as constipation and alternating bowel with the increased permeability to lactulose
Tomova (2020 [25]	63 children with ASD and 27 control non-autistic children,	Calprotectin	ELISA	• ↑ Alpha diversity in autistic children at the phylum level • Significantly more present in samples of children with ASD: *Lactobacillus, Aerococcus, Burkholderia, Desulfohalobium, Desulfovibrio, Oxalobacter, Pseudocitrobacter,* and *Youngiibacter*	• Positive correlation of *Clostridium* strains with GI score and fecal calprotectin • Positive correlation of *Youngiibacter* and social interaction and repetitive score of ADI-R and GI manifestations • Correlation of *Nitriliruptor* with behavioral scores in ADOS-2 social affect domain, reciprocal social interaction domain, and total score • Correlation of *Methanomicrobiales* with social interaction, reciprocal and social interaction, and total raw score of ADOS-2 • Correlation of GI score with *Oxalobacter* and *Desulfohalobium* • Negative correlation of *Desulfohalobium,* with BMI
Laghi (2021) [26]	80 ASD preschoolers	Calprotectin	ELISA	• Negative correlation of *Akkermansia muciniphila* with intermediate fecal calprotectin levels • Positive correlation of *Prevotella* levels of calprotectin higher than 200 μg/g	• No differences between the median concentration of fecal calprotectin in patients with and without GI symptoms, even with consideration of age-based stratification of children • No association between ADOS and any bacterial groups • ↑ Sutterella and *Bifidobacteria* and ↓ Prevotella in patients with GI symptoms
Chamtouri (2023) [27]	28 ASD, 18 age-matched siblings, and 28 age- and sex-matched unrelated children	SCFA (↑Propionic and valeric acids in autistic patients at lower ages)	Gas chromatography	• *Bifidobacterium* and *Collinsella* occurring in younger autistic children which tend to be attenuated at older ages • Positive correlation of ↑ *Coriobacteriaceae* in autistic patients with SCFA • Negative correlation of acetate and *Veillonellaceae, Oscillospiraceae, Christensenellaceae, Eubacterium coprostanoligenes_*group*, Candidatus Gastranaerophilales,* and *Oscillospirales_UCG-010* • Negative correlation of butyric acid with *Eubacterium coprostanoligenes*_group • Positive correlation of isobutyric and isovaleric with *Peptostreptococaceae, Eggerthellaceae, Oscillospiraceae, Methanobacteriaceae, Christensenellaceae, Akkermansiaceae, Clostridia_UCG-014, Rikenellaceae, Anaerovoracaceae, Oscillospirales_UCG-010,* and *Actinomycetaceae* and negative correlation with *Bifidobacteriaceae, Lactobacillaceae, Pasteurellaceae,* and *Butyricocaceae* • Positive correlation of valeric acid with *Atopobiaceae, Peptostreptococaceae, Eggerthellaceae, Methanobacteriaceae, Erysipelotrichaceae, Akkermansiaceae*, and *Actinomycetaceae* and negative correlation with *Butyricicocaceae* • Caproic acid correlated positively with *Peptostreptococcaceae* and *Bacilli_RF39* and negatively with *Butyricicocaceae*	• Association of *↓ Bifidobacterium* and ↑ *Thermodesulfobacteriota* with severe autism • No significant differences between disease severity (CARS) and absolute levels of SCFA, except for the molar proportions of isobutyric and isovaleric
Liu (2019) [28]	30 autistic subjects and 20 controls	SCFA (↓ Acetate and butyrate and ↑ fecal valeric acid in ASD subjects)	Liquid chromatography	• ↓ Butyrate-producing taxa (*Ruminococcaceae, Eubacterium, Lachnospiraceae,* and *Erysipelotrichaceae*) and ↑ valeric acid-associated bacteria *(Acidobacteria*) in autistic patients	Enriched *Fusobacterium*, *Barnesiella*, *Coprobacter,* and valeric acid-associated bacteria (*Actinomycetaceae*) and reduced butyrate-producing taxa in constipated autistic subjects
Kang (2018) [29]	23 autistic children and 21 controls	SCFA (No differences between propionate and butyrate between control and ASD patients)	NMR spectroscopy	↓ *Prevotella, Coprococcus Faecalibacterium (F. prausnitzii*,a butyrate producer*)* and *Haemophilus (H. parainfluenzae)* in ASD patients	Positive relation between GI symptoms and ATEC
De Angelis (2013) [30]	10 autistic children, 10 PDD-NOS, and 10 healthy controls	SCFA	Gas chromatography	• Positive correlation between the level of *Clostridium* species and the amount of methyl esters (butanoic acid methyl ester, acetic acid methyl ester, and pentanoic acid methyl ester) and indoles • Positive correlation of *Faecalibacterium* and *Ruminococcus* and *Bifidobacterium* genera with total SCFA and *Bacteroides* genus with propionic acid	N/A
Deng (2022) [31]	45 autistic children and 45 typically developing ones	SCFA (↑ propionic acid, butyric acid, and valeric acid in the ASD group)	Gas chromatography/mass spectrometry	• Association between SCFAs and *Hydrogenedentes, Elusimicrobia, Methylomirabilota, Crenarchaeota, MBNT15, Halobacteria, Chloroflexi, Actinobacteria,* and *Campylobacter*	• Positive correlation of alpha diversity with eating behaviors in contrast to *Bacteridota* • Higher diversity in the ASD patients with GI symptoms group • Enrichedd *Clostridiales, Clostridiaceae, Roseburia intestinalis, Megamonas, Selenomonadaceae,* and *Eubacterium eligens* groups in the ASD with GI symptoms group along with *Oxalobacteraceae, Gamaproteobacteria, Burkholderiates, Agathobacter,* and *Proteobacteria* at phylum levels

## S100B

Protein and peptide-based biomarkers have been the subject of some research for early diagnosis of ASD [7]. The S100 calcium-binding protein beta subunit (S100B) is expressed in astrocytes and other extra-neural cells, including enteric glial cells (EGCs). It affects neurons depending on the concentration, which can be trophic up to a few nanomolar doses and toxic at micromolar levels. Extracellular protein S100B contributes considerably to neuroinflammation by acting synergistically with pro-inflammatory cytokines and, at higher concentrations, acting as a cytokine [32]. Despite the existing conflict [33–35], multiple studies have found a significant elevation of S100B in autistic individuals compared to healthy cases, supporting the possible role of this factor in the etiology and development of ASD [36–40]. The source of this elevated S100B concentration in the periphery can be injured neurons or EGCs. In a recent study, the correlation between plasma S100B levels and fecal concentrations of calprotectin (an objective marker of GI inflammation status) revealed that not only brain astrocytes but also EGCs might be involved in the pathophysiology of autism [41]. One hypothesis says that the alternation of enteric glial-derived S100B expression in autistic patients can result from changed microbiota, disruption of the intestinal barrier, and even pathogenic bacteria, altogether inducing intestinal inflammation and converting EGCs to reactive EGCs [38]. Another *in vivo* study in mice showed that gut microbiota biodiversity increases with S100B levels or oral administration. Firmicutes phylum, including *Lactobacillus* and Bacteroidetes, including *Barnesiella* and *Butyricimonas* spp, are affected by S100B levels [42]. However, higher levels of *Bacteroidetes* and lower levels of *Firmicutes* were observed in a group of children with autism [43]. No study clarified the correlation between gut microbial alternation and the effect of probiotic use and S100B levels in autistic patients. Studies can be directed to know the effects of probiotic administration as a manipulative factor of gut microbiota on the levels of S100B in ASD patients. Furthermore, it is suggested that S100B can be investigated as a potential biomarker both in the diagnosis and treatment of autism.

## Brain-derived Neurotrophic Factor

BDNF is a protein member of the nerve growth factor family (neurotrophins). BDNF has a key role in both the pre-synaptic site (modulates neurotransmitter release) and post-synaptic site (augments the function of ion channels), so it generally contributes to affect neuroplasticity and, thereby, behavior-related conditions [44]. Abnormal levels of BDNF were seen in a wide range of neurological diseases, including schizophrenia, depression, and even autism [45]. According to recent studies, altered BDNF levels were observed in ASD patients compared to the controls, revealing that BDNF might play a role in autism pathophysiology [46–50]. A relatively higher level of BDNF was seen in mild phenotypes compared to severe autism, emphasizing the probable protective function of this factor [51]. Downregulation of the BDNF in the antiapoptotic signaling pathway in the brains of autistic individuals is one of the possible underlying mechanisms in the pathophysiology of autism [52]. The reduction of BDNF expression as a neuroprotective agent can be caused by raised inflammatory factors, including IL-1β and TNF; therefore, it may have a negative regulatory role in neuroinflammation [53, 54]. The dysbiotic gut microbiota in autistic patients may contribute to this inflammatory condition through immune dysregulation and the release of inflammatory factors such as IL-1β, which crosses the BBB [55]. Animal studies showed that BDNF has been lower in germ-free mice's cortex and hippocampus [56]. Probiotic administration in these sterile mice also resulted in partial and complete normalization of behavior and BDNF levels, respectively. It has also been suggested that probiotics, specifically a combination of the *Lactobacillus* and *Bifidobacterium* genera, may be effective in increasing BDNF levels and improving mental health parameters in patients with depression and neurological disorders [57, 58]. Balance of fecal *Clostridium spp.* and normal BDNF expression were both achieved through fecal microbiota transplantation or *Bifidobacterium* treatment in an animal model of autism [21]. In another rat model study, *Lactobacillus* supplementation could increase BDNF levels and attenuate behavioral anomalies [59]. Regarding these relations, further studies are needed to know if the induction and modification of microbial alteration in the gut of autistic patients can be monitored and controlled by BDNF levels.

## RANTES AND Eotaxin

Regulated upon Activation, Normal T Cell Expressed and Secreted, RANTES (CCL5), and eotaxin (CCL11) are pro-inflammatory chemokines released by a variety of cells, including blood cells, fibroblasts, endothelium, epithelium, neurons, and glial cells [60, 61]. RANTES [60, 62–66] and eotaxin [65–68] plasma levels are considerably higher in autistic children. Since RANTES and eotaxin act as pro-inflammatory mediators, their rise implies that both play a neuroinflammatory role in ASD [60, 61, 69, 70]. Although Shen et al. [63] reported no significant correlations between RANTES or eotaxin and behavioral patterns of ASD, Han et al. [64, 65] and Hu et al. [67] found RANTES and eotaxin related to ASD, respectively. Besides, other studies demonstrated that the rise of both factors is ASD related [66]. Moreover, gut microbiota seems to induce RANTES-mediated inflammation [71–73]. Earlier studies uncovered the NOD‐like receptor family pyrin domain containing 6–gut microbiota axis and subsequent IL-6 and TNF release as one possible connection of gut microbiota dysbiosis with RANTES-mediated immune dysregulation [74, 75]. Concerning the expression of gene encoding, it has been found that gut microbiota can manipulate eotaxin expression levels [76]. On this matter, antibiotic-treated mice had an altered microbiome with elevated eotaxin and different structures in their microglia [77]. Also, it has been found that mice's eotaxin levels changed after fecal microbiota transfer [78]. Regarding gut microbiota and ASD relation, modified anxiety-like and repetitive behaviors were observed while the levels of RANTES and eotaxin were improved through gut microbiota transplant in ASD mice. These results showed that RANTES and eotaxin play important roles in CNS synaptic transmission and development, and their levels are associated with the structure of microbiota in mice [22]. *Clostridiaceae*, *Erysipelotrichaceae Prevotella families*, *Candidatus Arthromitus*, and *Proteus* genus were found to be inversely associated with the level of RANTES and eotaxin [22]. *In-vivo* topical and oral probiotic administrations have reported a connection of RANTES with strains *Lactobacillus paracasei SGL 04, Lactobacillus plantarum SGL 07, Lactobacillus fermentum SGL 10, and Lactobacillus brevis SGL 12 lysates,* and *Lactobacillus rhamnosus GG* [79, 80]. Similarly, Probiotics containing *Lactobacillus acidophilus, Lactobacillus rhamnosus GG,* and *Bifidobacterium* also changed eotaxin gene expression in an animal [81]. Overall, the important findings implied from these studies suggest a potential mechanism of gut microbiota in ASD pathogenesis and severity through inflammatory factors of RANTES and eotaxin.

## GM-CSF

The cytokine granulocyte–macrophage colony-stimulating factor (GM-CSF) drives many aspects of myeloid hemopoietic cell biology, including survival, proliferation, differentiation, and functional activity. It also affects the immune system through dendritic and T-cell functions [82, 83]. GM-CSF triggers chronic inflammation in the CNS and acts as a neuronal growth factor to stimulate neuronal and glial differentiation [82–84].

Although some earlier studies presented a low GM-CSF level in autistic patients [85, 86], higher levels of GM-CSF were found consequently in the brains of ASD patients [70, 82–84]. Perroud et al. reported higher levels of GM-CSF- IL-1α, TNF-α, and interferon-α among ASD children experiencing co-morbid GI symptoms [87]. The changes in GM-CSF levels in ASD can indicate that an inflammatory process may be involved in developmental and neuroimmune impairment [83]. Results of co-culture experiments by Takada et al. are the first to show that GM-CSF-induced macrophages inhibit the dendritic outgrowth of neurons in autistic individuals. This phenomenon is mediated through the secretion of pro-inflammatory cytokines, IL-1α and TNF-α, and may lead to more severe behavioral effects [88].

Interestingly, GM-CSF levels vary with alterations in gut microbiota [89–92] and mostly with IL-17a, a cytokine that correlated with the severity of behavioral symptoms in individuals with ASD [89, 90]. Different species of gut bacteria have been linked to GM-CSF, including *Parabacteroide, Prevotella, Streptococcus, Clostridium, Lactobacillus reuteri, Lactobacillus crispatus Enterococcus faecalis, Blautia, Butyricimonass, Roseburia, Anaerotruncus,* and *Blautia* [89, 92]. An important finding showed that gut microbiota-derived metabolites like SCFAs may alter GM-CSF levels [90]. Within a study, GM-CSF as a neuroimmune factor was increased with the administration of probiotics containing *Bifidobacterium longum, Lactobacillus delbrueckii bulgaricus,* and *Streptococcus thermophilus* [93]. Altogether, the change of GM-CSF neuroinflammatory factors by gut microbiota alteration provides insight into the mechanism of pathogenesis in this way in ASD patients.

## HMGB-1

The high mobility group box 1 protein (HMGB-1) is one of the most abundant members of the HMGB protein family and has many potential roles [94]. It has a key role in DNA regulatory activities as a nuclear protein [95]. As an extracellular factor, it is actively released when immune cells respond to an inflammatory condition [96] and also passively released by necrotic or damaged cells [95]. HMGB1 has numerous membrane receptors called pathogen recognition receptors, TLR4, TLR9, and receptors for advanced glycation end products (RAGE) are the dominant ones. Through its interactions with these receptors, HMGB1 promotes inflammation in cells [97]. HMGB1 can cross the blood–brain barrier, promote neurite outgrowth and cell migration, or mediate neuroinflammation after injury [98].

It has been understood that plasma levels of HMGB-1 can elevate in ASD patients [99] and positively correlated with the severity of autism [100]. Another effective inflammatory molecule, the epidermal growth factor receptor, was considered to be related to symptom severity in children with autism, and the HMGB1 level seems to correlate with that [101, 102]. Interestingly, higher HMGB1 levels are found to be associated with higher GI dysfunctions in individuals with autism, which can imply an intestinal concept of pathogenesis [23, 103]. It is similarly studied that fecal levels of HMGB1 were correlated with GI sign severity in ASD children, which regards ASD-related dysbiosis [23]. Microbiome dysbiosis accompanied by intestinal inflammation can lead to the activation of monocytes, upregulating HMGB1 excretion for a pro-inflammatory feedback loop [104].

Higher levels of HMGB1 and TLR4 have also been reported to be associated with autistic-like behaviors in mice, possibly through activation of the HMGB1/TLR4 signaling cascade [105]. Serum levels of TLR4 were elevated in ASD children and positively associated with their hyperactivity scores [106]. Activation of the HMGB1/RAGE/TLR4 axis leads to leukocyte infiltration into nerve cells and results in persistent CNS inflammation. It is suggested that neuroinflammation is strongly related to ASD occurrence [107] through activating the inflammasome system as a mechanism [108]. In addition, it is described that HMGB1 can bind to endogenous secretory RAGE, resulting in a decline in plasma RAGE levels. This may contribute to the pathophysiology of autism by interfering with neuropeptide oxytocin transport from the periphery to the brain [109].

The effect of probiotics and gut microbiota alteration on HMGB1 levels in ASD patients can strengthen the idea and can be further studied. HMGB1 might play a key role in ASD pathogenesis through neuroinflammation and can conduct treatment strategies. However, it is a highly potential factor in the pathophysiology of autism, not precisely clarified, and more research is needed.

## Osteopontin

Osteopontin (OPN) is both a soluble proinflammatory cytokine with a well-established role in autoimmune neuroinflammatory diseases and a component of the non-collagenous bone matrix that controls biomineralization in bone tissue [110]. Depending on its location and context, OPN is involved in local inflammation, cell adhesion, immune response, chemotaxis, and protection from apoptosis [111]. Heilmann et al. hypnotized that OPN can activate the immune system, reduce tissue damage, and stimulate mucosal repair during acute inflammation while promoting the Th1 response and strengthening inflammation under chronic circumstances [112].

OPN has been related to the pathogenesis of neuropsychological disorders like multiple sclerosis and Alzheimer's disease [110, 113]. Expression of secreted phosphoprotein 1 and its encoded protein OPN by CD11c + cells were associated with cognitive impairment and common neuropathologies in Alzheimer’s disease [114]. Studies on OPN levels in autistic patients are limited. However, Al-ayadhi and Mostafa [111] found an association between elevated serum levels of OPN and disease severity, indicating the role of OPN in neuroinflammation and the development of brain-specific auto-antibodies. Their findings can support the idea of OPN as an important neuroinflammation factor in the mechanism of ASD.

The possible interaction of OPN with gut microbiota has been discussed in metabolic disorders [115]. However, the role of OPN is not yet studied in association with gut microbiota in neurological disorders, especially in ASD patients, and can be a potential target for future studies. The finding of alterations in specific strains of gut microbiota connected to OPN and symptoms of ASD may help to improve diet, treatment methods, and probiotic supplements.

## Calprotectin

Calprotectin is a protein that binds to calcium and is mainly found in neutrophils, which are white blood cells that increase when inflammation and cell damage occur. Calprotectin in stool can indicate intestinal inflammation and serve as a biomarker [116]. Considering the possible role of gut inflammation in the development of ASD, a number of research have studied the association of calprotectin levels in ASD patients, but their results were inconsistent. Some reports show that ASD patients and their relatives may have higher calprotectin levels than control groups [41, 117]. Interestingly, Babinská et al. found that calprotectin levels of ASD individuals were significantly related to all domains of autism diagnostic interview-revised, which measures social interaction, communication, and restricted and repetitive behaviors [41].

Similarly, Iovene et al. reported a significant correlation between autism severity, calprotectin level, and *Clostridium* spp—abundance [24]. Contrarily, Azouz et al. found no relation between calprotectin and disease severity, though they revealed a moderate correlation between calprotectin and GI symptoms [118]. Tomova et al. also revealed a positive correlation between *Costridiacae* bacteria, the severity of GI manifestations, and behavioral symptoms of ASD children. Calprotectin levels were also moderately correlated with higher expression of macrophage inflammatory protein 1β, which was associated with communication subscale and total score of autism diagnostic observation schedule, indicating that it may play a role in microbial-neuronal cross-talk [25]. Unlikely, some investigations found no statistically significant difference in calprotectin levels between ASD patients and controls [119–122] and, consequently, no appreciable variation in calprotectin levels of ASD patients with and without GI symptoms.

Studies on probiotic effects on calprotectin levels and autism are limited in the literature. Laghi et al. showed that greater calprotectin levels were associated with more *Prevotella* and fewer *Akkermansia* bacteria in the gut, indicating these bacteria may have inflammatory or protective effects, respectively [26]. However, Santocchi et al. found probiotic therapy, including eight strains of *Streptococcus*, *Bifidobacterium*, and *Lactobacillus*, to have a favorable impact on adaptive functioning in ASD patients but no discernible impact on calprotectin levels with or without GI symptoms [123]. This indicates that the probiotic effect on autistic patients is more complex than the reduction of gut inflammation, and the role of calprotectin as a probable neuroinflammatory mediator should be more studied.

Overall, the heterogeneities of calprotectin studies could be due to the diversity of trialed individuals, the accuracy of the used methods, and insufficient simultaneous studies of microbiota alterations and calprotectin. However, it is still possible to understand that host-microbiota dysbiosis and inflammation-induced calprotectin trigger neuroinflammatory mechanisms that cause autistic aspects.

## Gut Microbiota Metabolites and ASD

Many gut microbiota-derived metabolites are highlighted in ASD, such as complex polysaccharides or metabolic amino acids, which can be neurotransmitters [124]. Several of them have been recently discussed as early diagnostic biomarkers of ASD [7]. One significant group of metabolites through which gut microbiota regulates the host physiology is short-chain fatty acids, which primarily constitute acetate (AA), butyrate (BTA), and propionate (PPA).

The genera *Prevotella*, *Bifidobacterium*, and *Ruminococcus* are the primary producers of acetate [125], the most prevalent SCFA, which is reported to be decreased in ASD [29, 126]. BTA is mainly produced by the *Firmicutes* phylum, more precisely by *Lachnospiraceae* and *Ruminococcaceae* families [127], and PPA is synthesized by the *Bacteroidetes* phylum (including *Bacteroides* and *Prevotella*) and *Firmicutes* phylum (including *Roseburia*, *Blautia* and *Coprococcus*) [128]. However, alongside *Bacteroides*, the elevated level of PPA is associated with increased *Clostridium* and *Desulfovibrio* species in autistic individuals [129]. Also, a study on autistic children revealed lower *Bifidobacterium* and higher PPA levels, both of which attenuated at older ages [27].

Unlike some studies [28, 29, 126], others reported higher levels of AA, PPA, and BTA in autistic patients compared to control groups [27, 30, 31, 130, 131]. These gut microbiota-related SCFAs exhibit conflicting pro-inflammatory and anti-inflammatory effects in the host's inflammatory response, possibly due to the differences in binding receptors and local concentrations [132]. Some animal studies revealed that supplementation with the microbial metabolites AA and BTA could reverse the social behavioral phenotypes [133–136]. In contrast, intracerebroventricular injection of PPA in rat brains has induced ASD-like symptoms, including reactive gliosis [137]. It has been understood that PPA can lead to gliosis, disturbed neuro-circuitry, and neuroinflammatory response through modulation of the PTEN/AKT pathway in ASD [138]. As the finding data regarding SCFA levels in autistic patients are inconsistent and yet to be studied [7, 28, 124, 139], additional research is required to verify the potential role of SCFAs in the pathophysiology of ASD. They might be considered as neuroinflammatory biomarkers and indicators of gut microbiota modification in autism patients.

## MicroRNAs and ASD

Over 60% of human genes are controlled by microRNAs (miRNAs), small, non-coding RNAs of around 18–24 nucleotides that function as epigenetic regulators. MiRNAs modify brain plasticity and neuronal development, and their dysregulation causes a broad spectrum of neurological impairments, including ASD [140–144]. The importance of miRNAs as regulators of numerous cellular and physiological processes, including hematopoiesis, immune reactions, and inflammation, is well-established [145]. Additionally, miRNAs are affected by host-microbiota interactions and play a key role in dysbiosis and induced inflammations [146–149]. An intensive study found over-expressed miRNAs in ASD and their possible role in impaired neurodevelopment through dysregulated inflammatory genes [150]. Besides, several studies have identified that miRNAs directly and indirectly activate inflammasomes through their interaction with 3'-UTR genes that modulate inflammasome expression [151].

In detail, animal studies suggest that an increase or decrease of miR-146a can be a potential cause of ASD [152]. A clinical study of the postnatal period compared miRNAs of ASD and healthy controls and confirmed miR-146a as the most dysregulated miRNA in ASD [152]. Using *in vitro* models and postmortem human brain tissues, another study also found that miR-146a overexpression in the brains of ASD patients is detectable as early as childhood [153]. The changes in Gut microbiota-host interaction could induce miR-146a and consequently promote neuroinflammatory pathways [154]. It is highlighted that miR-146a-induced nuclear factor kappa-B augments the inflammation signaling pathway in the gut-brain axis. It has been shown that *Bacteroides fragilis, Lactobacillus rhamnosus GG, Lactobacillus acidophilus*, *Lactobacillus delbrueckii Bulgaricus*, and *Escherichia coli Nissle 1917* were linked to miR-146a expression [149, 154, 155]. Another research indicates that miR-146a is essential for certain inflammatory cytokine expression and that its absence in the brain leads to an overall compensatory upregulation of miR-155. Enhanced protein carbonylation and decreased cysteine thiol levels were additional indicators of this elevated neuroinflammatory flux due to an upsurge in oxidative stress mediators [156].

Several studies have identified miR-146a and miR-155 to various pathologic conditions indicated by chronic inflammation [157]. A possible explanation is that gut-derived toxins, such as LPS, capable of traversing the blood–brain barrier and are in systemic circulation, can potentially activate the NF-kB-miRNA-146a-miRNA-155 signaling pathway. This pathway would then transmit pathogenic signals originating from the microbiome to the brain, which might disturb the innate immune reactions and lead to neuroinflammatory conditions [158]. MiR-155 could also be altered by gut microbiota dysbiosis [159]. One study added evidence of increased miR-155 expression in the amygdala, frontal cortex, and cerebellum of children with ASD [62]. miRNA-155 is involved in TLR activation by bacterial lipopolysaccharides, activation of tumor necrosis factor-alpha and IL-6, and regulation of suppressor of cytokine signaling 1 on dendritic cells. These activities, alongside the variation with microbiota dysbiosis, can give a candidate role to miRNA-155 in the neuroinflammatory mechanism of the gut-brain axis and ASD [152, 159]. Earlier studies identified probiotics of *Lactobacillus fermentum, Lactobacillus salivarius, Lactobacillus rhamnosus GG, Lactobacillus acidophilus, Lactobacillus delbrueckii, Bifidobacterium bifidum, and E coli Nissle 1917* could change the level of miR-155 [149, 155, 159, 160].

Moreover, studies found upregulated miR-181 in ASD patients, expected to impact the ASD-related *neurexin 1* gene [152, 161, 162]. Neuroinflammation and immunological dysregulation are two of the many physiological processes linked to the miR-181 family [163–165]. On the other hand, some studies show that gut microbiota could regulate miR-181 in mice [148, 166, 167]. It has also been revealed that *Lactobacillus rhamnosus* and *Lactobacillus delbrueckii* probiotics affect the miR-181a expression in inflammatory diseases [160]. Additionally, metabolites derived from gut microbiota could affect miR-181 expression in different states [148]. Altogether, these pieces of evidence strengthen the argument about the possible miR-mediated role of gut microbiota through the neuroinflammatory process in ASD.

## Probiotics and ASD

Living microorganisms known as probiotics can influence host health through various mechanisms. According to recent research, they can be used as a therapeutic tool to treat ASD by restoring a healthy balance in the gut microbiota, adjusting the levels of neurotransmitters in the tissues, and reducing inflammation in the gut [168, 169].

Animal models revealed that probiotic supply considerably modified the social and emotional behaviors of the rats as well as blood levels of cytokines like IL-6, IL-17a, and IL-10 [59, 170, 171]. On the other hand, only a few trials assessed the impact of probiotics on ASD with the aspect of inflammatory modulation and immune system regulation (Table 2). Sanctuary et al. evaluated the use of *Bifidobacterium infantis* in combination with a bovine colostrum product in autistic children. Some patients revealed lower frequency of GI symptoms and aberrant behavior, possibly due to a reduction in TNF-α and IL-13 [172]. Tomova et al. also showed a strong correlation between fecal levels of TNF-α and the severity of autism, indicating the possible involvement of GI inflammation and permeability in ASD through inflammatory pathways. They could significantly decrease the TNF-α levels in the feces of autistic children through probiotic supplementation involving strains of *Lactobacillus*, *Bifidobacteria*, and *Streptococcus* [173]. However, Santocchi et al. found the plasma levels of plasma inflammatory biomarkers, including TNF-α, IL-6, leptin, and plasminogen activator inhibitor 1, and fecal calprotectin contrarily unaffected by the probiotic treatment, involving the same genera as Tomova et al.… Nevertheless, there is a greater improvement in some GI symptoms, adaptive functioning, and sensory profiles in the group treated with probiotics compared to placebo in the subgroup of autistic children with GI problems [123]. Similarly, using strains of *Bifidobacterium* and *Lactobacillus* alongside an oligosaccharide could improve disease severity and GI problems in autistic children [174].

**Table 2:** Trials on probiotics effects in inflammation and ASD management

Author (year)	Study Design, Duration	Sample Size (Intervention/ Control)	Age (years + SD)	Probiotic	Microbial Alternation	Immunomodulation findings	Clinical Improvement	Scale
Schmitt (2023) [175]	Double-blinded, crossover RCT, 28 days	8/ 7	Range: 15- 45	SB-121, a combination of *Limosilactobacillus reuteri*, Sephadex® (dextran microparticles), and maltose	N/A	No relevant changes in the plasma TNF-α and HS-CRP, and fecal calprotectin and lactoferrin	Improvements in adaptive behavior and social preference	Vineland-3 adaptive behavior composite score and eye tracking
Kong (2021) [176]	Double-blinded, randomized, placebo-controlled, two-stage pilot trial, 28 weeks	14/ 13	10.3	*Lactobacillus plantarum* PS128	• The absolute change (V3-V1) in *Eubacterium hallii* group abundance in the combination therapy group is positively correlated with the baseline SRS cognition score. • The absolute change (V3-V1) in *Rikenelaceae*, *Alistipes*, *Christensenellaceae R7*, and *Ruminococcaceae UCG-002* in the combination therapy group positively correlated with the ABC stereotypic behavior score at baseline. • *Christensenellaceae R7* and *Ruminococcaceae UCG-002* are found only in the combination treatment group.	↓ IL-1β	Improvement in the total ABC, stereotypic behavior, and SRS cognition score with no significant differences in the total scores or subscales of the ABC and SRS, ↓CGI score	ABC, SRS, and CGI
Santocchi (2020) [123]	Double-blinded RCT, 6 months	42/ 43	4.2	DSF^2^, consisting of 1 strain of *Streptococcus*, 3 strains of *Bifidobacterium*, and 4 strains of *Lactobacillus*	N/A	No statistically significant changes in plasma levels of IL-6, TNF-α, PAI-1, and fecal calprotectin	No differences in total ADOS-CSS scores, but ↓ total ADOS-CSS scores and ↓ social-affect ADOS-CSS in patients without GI symptoms, Improvement in GI symptoms, adaptive functioning, and sensory profiles in patients with GI problems	Mainly ADOS-CSS, CBCL, and 6-GSI
Wang (2020) [174]	RCT, 12 months	26/ 24	4.4	4 strains of *Bifidobacterium infantis Bi-26, Bifidobacterium lactis BL-04, Lactobacillus* *Rhamnosus HN001, and Lactobacillus paracasei LPC-37+* Fructo-oligosaccharide	↑ *Bifidobacteriales and B. longum*, ↓ *Clostridium*	↑ SCFAs	↓ Autism severity, especially hyper-serotonergic state and dopamine metabolism disorder, and GI symptoms	ATEC, 6-GSI
Sanctuary (2019) [172]	Double-blinded, crossover RCT, 12 weeks	8/8 (prebiotic only^1^)	6.8 ± 2.4	*Bifidobacterium infantis* + Bovine colostrum product as a source of prebiotic oligosaccharides	No effect or an inconsistent effect on enterotype	↓ CD4+ cells producing intracellular IL-13, and CD8+ cells producing TNF-α	↓ Lethargy, ↓ Frequency of certain GI symptoms specifically pain with stooling and consistency	ABC score, QPGS-RIII and GIH questionnaire data, and parental reporting
Tomova (2015) [173]	Prospective, open-label, controlled, 4 months	10+ 9 nonautistic siblings/ 10	Range: 2-17	3 strains of *Lactobacillus,* 2 strains of *Bifidobacteria*, 1 strain of *Streptococcus*	↓ *Bifidobacteria, Lactobacillus,* and *Desulfovibrio spp.,* ↑*Bacteroidetes/Firmicutes* by ↓ *Firmicutes*	↓ Fecal TNF-α	↑ TNF-α levels linked to ↑ GI symptoms and ASD severity	CARS and ADI

1. The control groups were placebo except for the mentioned ones

2. DSF, marketed as Vivomixx® in EU, Visbiome® in USA, is a mixture containing 450 billion of *Streptococcus thermophilus, Bifidobacterium breve, Bifidobacterium longum, Bifidobacterium infantis, Lactobacillus acidophilus, Lactobacillus plantarum, Lactobacillus para-casei, Lactobacillus delbrueckii subsp. bulgaricus*

Abbreviations: SD, Standard Deviation; TNF-α, Tumor Necrosis Factor-alpha; HS-CRP, High Sensitivity C-Reactive Protein; IL, Interleukin; ABC, Aberrant Behavior Checklist; SRS, Social Responsiveness Scale; CGI, Clinical Global Impression; QPGS-RIII, Questionnaire on Pediatric Gastrointestinal Symptoms-Rome III Version; GIH, Gastrointestinal History; PAI-1, Plasminogen Activator Inhibitor-1; ADOS-CSS, Autism Diagnostic Observation Schedule - Calibrated Severity Score; CBCL, Child Behavior Check List; GSI, Gastrointestinal Severity Index; SCFA, Short- Chain Fatty Acid; ATEC, Autism Treatment Evaluation Checklist; CARS, Childhood Autism Rating Scale; ADI, Autism Diagnostic Interview

*Limosilactobacillus* genus can also lead to improvement in adaptive symptoms of ASD [175, 177]. However, Schmitt et al. did not see any relevant changes in the plasma TNF-α and HS-CRP, fecal calprotectin, and lactoferrin with the use of this probiotic [175]. Synergic use of *Lactiplantibacillus plantarum* and oxytocin was also revealed to have an anti-inflammatory effect through the reduction of IL-1β [176]. The probiotic mixture containing five strains of *Bifidobacterium longum* with anti-inflammatory and high homeostatic intestinal activity, along with *Limosilactobacillus fermentum*, *Lactiplantibacillus plantarum*, and *Ligilactobacillus salivarius*, showed significantly alternation the diversity of gut microbiota. The species that are consistent with this formulation of probiotics were found in the feces of autistic children, including *Streptococcus thermophilus*, *Bifidobacterium longum*, *Limosilactobacillus fermentum*, and *Ligilactobacillus salivarius* [177].

There are no medicines indicated for the core deficits of ASD. Therefore, there is a substantial requirement for the creation of novel pharmacological approaches for patients with ASD. Overall, these findings support that probiotics may serve as a promising therapy due to their beneficial impact on symptoms of ASD. Considering the existing association between immune system dysfunction and behavioral abnormalities [178] and the possible impact of gut microbiota on ASD through inflammatory mediators, it is suggested that neuroinflammatory variables be examined during probiotic administration and the most effective formulation to alter them be determined.

## Conclusion

Given the complexity and lack of clarity surrounding the pathophysiology of ASD, research into the role of inflammatory mechanisms and immunological dysregulation has been raised in recent years. Dysregulation pathways in ASD may also be etiologically traced back to gut microbial alterations and host-microbiota dysbiosis. These changes have been associated with ASD symptoms and severities probably through the released metabolites, neural signaling pathway by BDNF, and neuroinflammatory biomarkers, including S100B, HMGB-1, OPN, miRNAs, RANTES, eotaxin, and GM-CSF. In this review, the role of mediators as a triggering mechanism and bridging cause between gut microbiota dysbiosis-induced inflammation from one side, and neuroinflammatory processes of CNS in autism from the other side is emphasized. Probiotics as an applicable therapeutic option to recover microbiota in ASD suggest the relevance of gut microbiota and potential beneficial impacts. However, further studies are essential to evaluate the efficacy of different probiotic formulations considering microbiota alteration types, coincidence neuroinflammatory mediators, intervention length, and autistic age and symptoms. In fact, many ideas have been proposed to explain ASD pathogenesis, but there is currently a lack of intensive immunological, neurochemical, and microbiota studies in the field. This approach can clinically explain the trajectory through microbiota alteration, related metabolites, neurological inflammatory mediators, and the CNS process of ASD. This constructed dogma can be used to create etiologic, diagnostic, prognostic, or therapeutic targets for ASD.

## Data Availability

Not applicable.

## Abbreviations

ASD: Autism spectrum disorder
SCFAs: Short-Chain Fatty Acids
BDNF: Brain-derived neurotrophic Factor
GI: Gastrointestinal
IL: Interleukin
TLR: Toll-like receptor
TNF: Tumor necrosis factor
S100B: S100 calcium-binding protein beta subunit
EGCs: Enteric glial cells
RANTES: Regulated upon Activation, Normal T Cell Expressed and Secreted
GM-CSF: Granulocyte–macrophage colony-stimulating factor
HMGB-1: High mobility group box 1 protein
OPN: Osteopontin
AA: Acetate
BTA: Butyrate
PPA: Propionate
miRNA: MicroRNA
